# Reply to Rubber Company's Chemical Advocate

**Published:** 1872-04

**Authors:** J. R. Walker

**Affiliations:** New Orleans, La.


### ARTICLE III.

### *Reply to the Rubber Company's Chemical Advocate.*

BY J. R. WALKER, D.D.S., NEW ORLEANS, LA.

EDITOR *American Journal of Dental Science:*—Some time ago, an article on the injurious effects of "Vulcanite Plates," appeared in the "American Chemist," from F. W. Clark, S. B., who claims to have been lecturing in the Boston Dental College.

As the positions taken, and statements made, vary so widely from known facts, and the article is so well calculated to mislead suffering humanity on the one hand, and to furnish an excuse to ignorant or unprincipled operators, on the other, I intended to reply through the "Chemist;" but as lack of time prevented my sending the reply at the proper time, and it has since appeared in your editorial department, I will reply, by your leave, through the same medium, and thus apply the antidote where the poison has gone.

In regard to the implied claim of the author, to be in possession of "what little is known on the subject," I will ask what likelihood there is, of a professional chemist having access to a sufficient number of persons wearing rubber plates, and examining them with sufficient care, to know anything about it practically, and especially to negative the assertions of those who *have seen* and *do know* of the fearful ravages of the *rubber disease*, which has become so common, that to report the cases, would occupy all the space of all the journals.

His claim, that the question is not settled, has no better foundation than, that the rubber advocates try to divert attention from the investigation of the rubber disease, by claiming that, "other plates make sore mouths."

He says, "again, thousands of Vulcanite plates are worn, and the cases of injury are comparatively rare." Now, sir, any one who has given honest, intelligent and close observation to this subject, knows the facts to be directly the reverse.

It is only *painful sore mouths* that are *rare*, while the injury is *universal*. I have seen the roof of the mouth so diseased as to present the appearance of a piece of half-decayed beef, cut across the grain; or, lines of inflammation extending to the throat, and there producing stomato-gastric derangement, causing nausea, loss of appetite, loss of flesh, and general debility; and all this without the cause being suspected by the sufferer, simply because there was no pain *under the plate.*

His other statements are equally erroneous and unfounded, and it would seem that a man, claiming to be a chemist, and making such wild assertions, must have unbounded confidence, either in his own invulnerability, or in general stupidity.

I confess to some difficulty in handling with patience an article, which, like the substance it endeavors to uphold, is brimful of poison, and which bears so plainly, the earmarks of the unprincipled rubber company.

The mechanical mixing of the sulphide with the rubber does not prevent its being dissolved in, and acting upon, the fluids of the mouth; and being thus mixed with all the nutriment, it permeates and poisons every fibre of the body, and this in such an insidious manner, and to the *superficial observer* with so little *apparent mouth disease*, that frequently constitutions are undermined and destroyed, and sometimes death ensues, before the real cause is ascertained.

It is true that the effect varies in different cases, owing to diosyncrasies, habits, etc., but among hundreds of cases of those wearing rubber plates, examined in the last three years, I have not found one free from the specific rubber disease in one form or another.

It is known to physicians, and should be to everybody, that the mucous membrane of the mouth is five times as sensitive to the action of drugs, as any other membrane of the body, and that the entire system can be more readily acted upon through that, than any other medium.

The red sulphide of mercury forming one third of the Vulcanite Plate, being in contact with this sensitive membrane, produces in *many* cases severe sore mouth, and in ALL CASES it *poisons* the *secretions*, and thus permeating the *entire system*, it deranges *all nutrition*, and attacks any weak point as of the throat, *lungs*, stomach, liver, etc.

To say that such a *poison*, under such circumstances, ought, *theoretically*, to be *inactive*, is as *absurd* as the assertion that it is practically so, is FALSE.

